# Efficacy and Safety of High-Dose Rifampicin in the Management of Tuberculosis Meningitis: A Systematic Review and Meta-Analysis

**DOI:** 10.7759/cureus.107718

**Published:** 2026-04-25

**Authors:** Syed Saad Ali, Wifag Medani Malik, Sandhya Narahari, Dani Badal Hayder, Naheemat Mofolasayo Salimon, Warda Batool Ali, Nidhi Reji, Roa Ali, Dipak Chaulagain, Aashan Ahmed, Abdulqadir J Nashwan

**Affiliations:** 1 Neurology, Pakistan Institute of Medical Sciences, Islamabad, PAK; 2 Hematology, Midland Metropolitan University Hospital, Birmingham, GBR; 3 Medicine, Siddhartha Medical College, Dr. NTR University of Health Sciences, Vijayawada, IND; 4 Medicine, University of Duhok, Duhok, IRQ; 5 Psychology, Teesside University, Middlesbrough, GBR; 6 Acute Medicine, Nottingham University Hospitals NHS Trust, Nottingham, GBR; 7 General Surgery, Rotherham General Hospital, The Rotherham NHS Foundation Trust, Rotherham, GBR; 8 Neurology, Royal Victoria Infirmary, Northumberland, GBR; 9 Neurosurgery, Uzhhorod National University, Uzhhorod, UKR; 10 Nursing and Midwifery Research, Hamad Medical Corporation, Doha, QAT

**Keywords:** high-dose therapy, pharmacokinetics, randomised controlled trials, rifampicin, tuberculosis meningitis

## Abstract

Tuberculosis meningitis (TBM) represents the most severe type of tuberculosis (TB), and it is characterized by high mortality and neurological complications. Rifampicin is a major part of the standard treatment of TB; however, conventional doses often achieve subtherapeutic levels in the central nervous system. High-dose rifampicin has been suggested to enhance drug exposure and, possibly, improve the clinical outcomes. This study is a systematic review and meta-analysis evaluating the effectiveness and safety of high-dose rifampicin compared with standard-dose rifampicin in the treatment of TBM. PubMed, the Cochrane Library, and ScienceDirect were searched to identify randomized controlled trials (RCTs) published between 2010 and 2026. The included studies involved adult patients with TBM receiving either high-dose or standard-dose rifampicin. Pharmacokinetic results and risk ratios (RR) were estimated using a random-effects model with data pooling to estimate mean differences (MD) and confidence intervals (CI). Eight RCTs were included. The maximum plasma concentration (Cmax) (MD 23.89 µg/mL; 95% CI (8.56, 39.21)) and total drug exposure (AUC(0-24)) (MD 148.66 µg·h/mL; 95% CI (19.38, 277.94)) were both found to be significantly higher with high-dose rifampicin compared to standard-dose rifampicin. No significant difference, however, was found in six-month mortality (RR 1.00; 95% CI (0.80, 1.26)). The risk of neurological adverse events was higher with high-dose rifampicin (RR 1.34), with other adverse events such as hepatotoxicity and hypersensitivity reactions being comparable between groups. In general, high-dose rifampicin enhances pharmacokinetic exposure in TBM, but with no significant reduction in mortality and a higher risk of neurological adverse events. More large-scale randomized trials are required to identify optimal dosing practices and improve clinical outcomes.

## Introduction and background

Tuberculosis (TB) remains a major global health concern, with an estimated 10.7 million new cases reported worldwide in 2024, predominantly affecting low- and middle-income countries [1]. Although pulmonary TB is the most prevalent type, tuberculosis meningitis (TBM) is a more serious form of the disease, leading to a high mortality rate and significant neurological impairment among survivors [2,3]. Despite timely diagnosis and treatment, mortality rates in TBM remain alarmingly high, ranging from 20% to 50%, highlighting the need for more effective therapeutic strategies [2].

The cornerstone of TBM management is standard TB treatment regimens that include rifampicin, isoniazid, pyrazinamide, and ethambutol [4]. Among these, rifampicin is especially important because of its strong bactericidal effect, mediated through inhibition of DNA-dependent RNA polymerase in *Mycobacterium tuberculosis*, thereby suppressing RNA synthesis and bacterial replication [5]. Nevertheless, the standard dose of rifampicin usually achieves subtherapeutic concentrations in the central nervous system (CNS), thereby limiting its ability to eliminate *M. tuberculosis* in the central nervous system [6]. This pharmacodynamic limitation can lead to adverse clinical outcomes even when following prescribed regimens [6].

High-dose rifampicin has been proposed as a possible measure to address these shortcomings [7]. Pharmacokinetic research suggests that higher doses of rifampicin result in increased plasma and cerebrospinal fluid (CSF) levels, which may improve bacterial clearance but not survival [7,8]. Several clinical trials have evaluated high-dose rifampicin in TBM. Although pharmacokinetic analyses indicate higher drug levels in plasma and CSF, randomized trials have not shown a definitive survival or treatment benefit, and potential harm in certain groups cannot be ruled out [8,9]. Nevertheless, safety, especially hepatotoxicity and other adverse events, is another factor to consider [10].

Although there has been increased interest in high-dose rifampicin therapy, studies have reported variable dosing strategies and inconsistent clinical outcomes, with limited evidence of improvement in mortality and treatment failure despite faster sputum conversion [11]. Moreover, the value of potential benefits versus risks is not clearly defined. Therefore, a comprehensive synthesis of available data is needed to provide better insight into the clinical impact of high-dose rifampicin in the treatment of TBM.

Thus, the purpose of this systematic review and meta-analysis was to assess the effectiveness and safety of high-dose rifampicin in patients with TBM by summarizing evidence from published clinical trials. In particular, the study aimed to compare clinical outcomes (i.e., mortality and treatment response) and adverse events between high-dose rifampicin regimens and standard-dose treatment.

## Review

This systematic review and meta-analysis were conducted according to Preferred Reporting Items for Systematic Reviews and Meta-Analyses (PRISMA) guidelines, and the study protocol was registered on PROSPERO (CRD420261341485) [12].

Search strategy

A detailed search strategy was developed for PubMed, The Cochrane Library, and Science Direct, as these were the three main databases we included. The search strategy combined keywords using 'AND' and 'OR'. For PubMed, we used '((((((Rifampin) OR (Rifampcin)) AND (Tuberculous Meningitis)) OR (TB meningitis)) OR (Meningitis)) AND (High Dose)) OR (Intensified Regimen)'. For Cochrane, we used ‘("rifampin"):ti,ab,kw OR (Rifampcin):ti,ab,kw AND ("tuberculous meningitis"):ti,ab,kw OR (TB meningitis):ti,ab,kw AND ("high dose"):ti,ab,kw’ and for science direct we used ‘(((Rifampin) OR (Rifampcin)) AND ((Tuberculous Meningitis) OR (TB meningitis) OR (Meningitis)) AND ((High Dose) OR (Intensified Regimen)))’. The search across all databases was restricted to randomized controlled trials (RCTs) and studies published from 2010 to March 2026.

Eligibility criteria

Studies were included if patients were aged over 18 years, had a diagnosis of TBM, and were treated with anti-tubercular therapy (ATT) for no more than three days before admission. Studies published in English with high-dose rifampin as the intervention, compared with standard-dose rifampin, using an RCT design, were included. Furthermore, studies had to report at least one of the primary outcomes to be included.

Studies were excluded if they were not RCTs or included patients under 18 years of age or if they included patients under 18 years of age with diagnoses other than tubercular meningitis. Additionally, studies involving pregnant patients or patients with liver function tests (LFTs) five times the normal level were excluded, as rifampin is a hepatotoxic drug, and impaired liver function can lead to improper metabolism of drugs. Studies that did not report any of the primary outcomes, as well as those reporting rifampin in combination with other drugs (excluding ATT), were also excluded.

Study screening, selection, and data extraction

We initially retrieved all articles from our databases into EndNote for duplicate removal. After removing duplicates, reviewers independently screened articles based on their titles and abstracts for inclusion. Articles that remained unexcluded at this stage were retrieved for full-text review. Thus, articles with full-text availability were screened against our eligibility criteria. Articles that fulfilled our selection criteria were included, while the rest were excluded. Additionally, the reference lists of the included articles were also searched for any relevant articles. In case of any confusion regarding the inclusion of an article, another reviewer was consulted to make an independent decision.

All included articles underwent data extraction, including author ID, age, male-to-female ratio, sample size, HIV status, intervention and comparator details, and outcomes.

Quality assessment and data synthesis

We assessed the quality of the included RCTs using the Cochrane Risk of Bias 2 (RoB2) tool [13]. Articles were given a final status of high risk, some concerns, or low risk. High-risk articles either had two domains with some concerns or one domain with high risk. Articles with some concerns had one domain with some concerns, with the rest of the domains being low risk. Similarly, articles with low risk had all domains showing low RoB.

RevMan was used to analyze the pooled data. For continuous outcomes, mean differences (MD) and standard deviations were reported under a random-effects model. For dichotomous variables, we used risk ratios (RR) under the Mantel-Haenszel random-effects model. We assessed heterogeneity using I², with heterogeneity below 40% considered insignificant. Heterogeneity above 75% was considered high, and was considered moderate for I² between 50%-75%. All outcomes were represented using forest plots with 95% confidence intervals (CI). A p-value below 0.05 was considered significant. Subgroup analysis could not be conducted due to limited data. However, a leave-one-out sensitivity analysis was carried out, along with the exclusion of high-risk studies. Similarly, publication bias could not be assessed because of the limited number of studies per outcome.

Results

**Figure 1:**
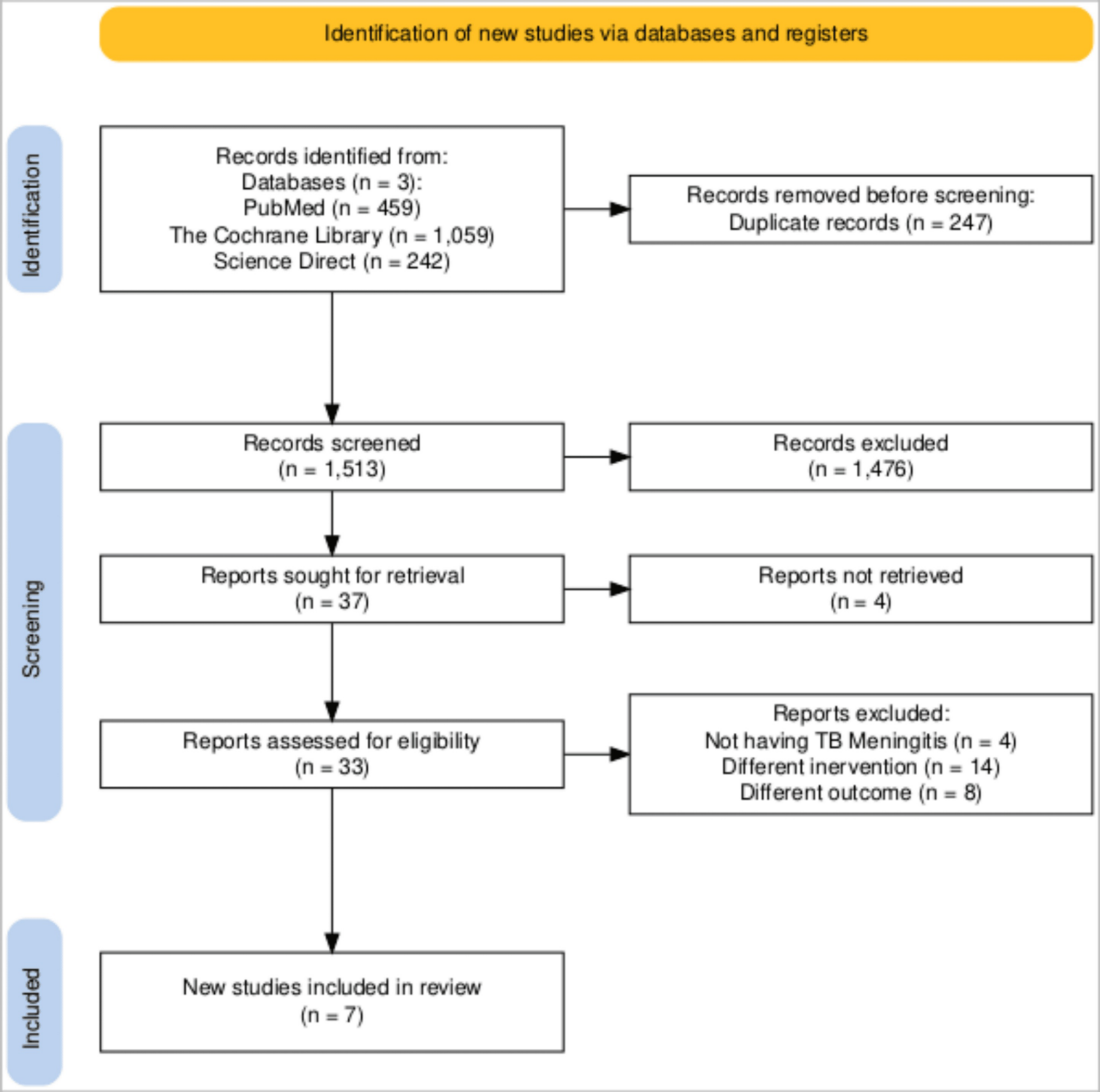
PRISMA flow diagram of study selection This figure illustrates the process of study identification, screening, eligibility assessment, and inclusion based on PRISMA guidelines for the meta-analysis. PRISMA: Preferred Reporting Items for Systematic Reviews and Meta-Analyses

We initially retrieved 1760 articles; 247 were marked as duplicates by EndNote and were manually excluded. A total of 1513 articles underwent title and abstract screening; only 37 relevant articles were identified, of which the full text of four could not be found. From the remaining 33 articles, seven were included, while the rest were excluded [7,9,14-19]. Four studies were excluded for not involving TBM, including one with participants aged below 18 years. Similarly, of the 14 studies excluded for using different interventions, some either did not use rifampin or used it in combination with other drugs, such as moxifloxacin or aspirin. Additionally, eight studies were excluded because they did not report any of the primary outcomes. The PRISMA flowchart summarizing the entire selection process is shown in Figure 1.

Characteristics of studies

Across all eight trials, baseline characteristics were evenly distributed between the higher-dose and standard-dose arms (Table 1). Males predominated in the study sample, which was mainly young to middle-aged (median ages 28-41.5 years). The majority of patients presented with (Medical Research Council) MRC Grade 2 or 3, especially in larger cohorts such as Heemskerk et al. and Meya et al. In several Southeast Asian cohorts (e.g., Ruslami et al. and Dian et al.), the median BMI values were close to the underweight criterion (<18.5 kg/m²). Overall, the consistency of baseline data across trials ensures a similar basis for assessing treatment outcomes and reflects good randomization.

**Table 1:** Baseline characteristics of included studies ^*^Values represent the number of female participants (n) with corresponding percentage. This table summarizes the baseline demographic and clinical characteristics of participants across the included studies, including age, gender distribution, weight, BMI, and disease severity (MRC grading) [7,9,15-19]. MRC: Medical Research Council; IQR: interquartile range

Study	Arm Type	N	Median Age (IQR)	Female % (n)	Median Weight (IQR)	Median BMI (IQR)	MRC Grade 1%/2%/3% (n)
Cresswell F (2021) [7]	Comparator (Control)	21	34.0 (27-36)	57.1% (12)^*^	50.0 (45-55)	-	9.5/57.1/33.3
-	Intervention (P/O 35 mg)	20	32.5 (26.5-38.5)	40% (8)^*^	50.5 (50-55)	-	5/80/15
Davis AG (2022) [16]	Comparator (Arm 1)	20	39.5 (34-48.5)	50% (10)^*^	-	-	-
-	Intervention (Arm 3)	16	41.5 (31.8-46)	37.5% (6)^*^	-	-	-
Heemskerk AD (2016) [18]	Comparator (Standard)	409	35 (30-47)	32% (131)^*^	-	-	39.1/43.5/17.4
-	Intervention (Intensified)	408	35 (29-45)	30.9% (126)^*^	-	-	38.7/43.9/17.4
Meya DB (2025) [9]	Comparator (Standard)	250	35 (28-45)	48.4% (121)	55 (45-62)	-	21.2/63.2/15.6
-	Intervention (Intensified)	249	38 (28-46)	40.6% (101)	54 (49-60)	-	20.1/59.4/20.5
Ruslami R (2013) [17]	Comparator (P/O 450 mg)	12	34 (19-47)	33% (4)^*^	50 (35-57)	18.0 (16-23.3)	-
-	Intervention (IV 600 mg)	10	29 (16-49)	60% (6)^*^	46 (34-54)	18.1 (15.1-21.6)	-
Wasserman S (2021) [19]	Comparator (P/O 10 mg)	17	38 (34-47)	47% (8)	64 (54-77)	25 (22-32)	59/41/0
-	Intervention (P/O 35 mg)	15	41 (36-45)	33% (5)	60 (53-80)	22 (20-23)	53/47/0
Yunivita V (2016) [14]	Comparator (P/O 750 mg)	11	33 (17-81)	45% (5)^*^	45 (40-54)	17.8 (16.5-22.8)	9/82/9
-	Intervention (600 mg IV)	10	31 (17-49)	40% (4)^*^	45 (40-60)	17.9 (14.7-24.1)	20/80/0
Dian S (2018) [15]	Comparator (P/O 10 mg)	20	28 (22.3-45.8)	40% (8)^*^	45 (40-47.3)	17.6 (15.4-19)	-
-	Intervention (P/O 30 mg)	20	33 (24.3-37.3)	40% (8)^*^	48.4 (41.6-54.7)	19.1 (16.2-20.2)	-

Quality assessment

**Figure 2:**
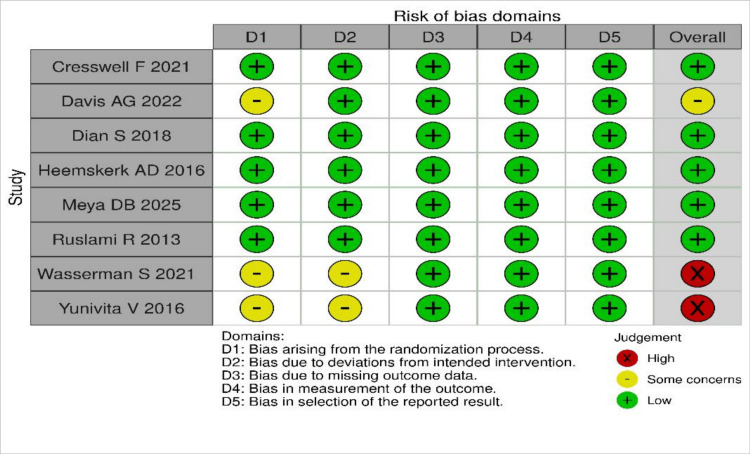
RoB assessment of included RCTs using the Cochrane RoB 2 tool RoB was evaluated across five domains: D1 (randomization process), D2 (deviations from intended interventions), D3 (missing outcome data), D4 (measurement of the outcome), and D5 (selection of the reported result). Each domain was categorized as "Low risk," "Some concerns," or "High risk" according to the Cochrane RoB 2 tool. The overall risk-of-bias judgment for each study was determined based on domain-level assessments [7,9,14-19]. RoB: risk of bias; RCT: randomized controlled trial

All studies included in this review had a low RoB, except the study by Davis AG et al., which had some concerns about blinding. Yunivita V et al. and Wasserman S et al. had a high RoB due to concerns about blinding and randomization. The summary of RoB is shown in Figure 2.

C-max and AUC (0-24) hours

**Figure 3:**
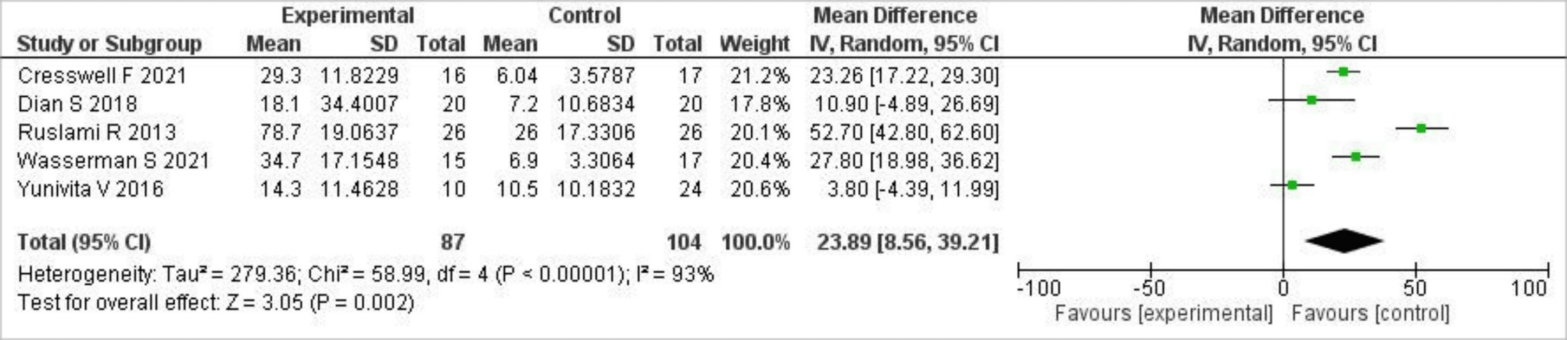
Forest plot of C-max This forest plot compares the C-max between high-dose and standard-dose rifampin groups, presented as MD with 95% CI [7,14,15,17,19]. MD: mean differences; CI: confidence intervals; C-max: maximum serum concentration

Our primary outcomes were the maximum serum concentration (C-max) achieved by the drugs and the area under the time-concentration curve (AUC) over 24 hours. For C-max, our analysis showed that high-dose rifampin achieved a higher serum C-max than standard-dose rifampin (MD 23.89; 95% CI (8.56, 39.21), P=0.002). However, a high level of statistical heterogeneity was observed (I²=93%). A forest plot below shows the results of C-max (Figure 3).

**Figure 4:**
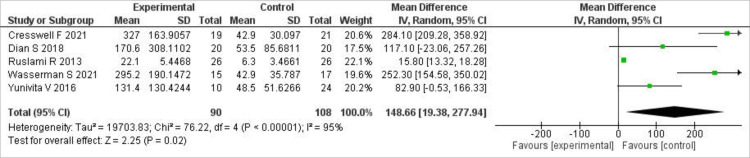
Forest plot of AUC (0-24) hours This figure shows the pooled analysis of AUC (0-24) hours between high-dose and standard-dose rifampin groups using MD with 95% CI [7,14,15,17,19]. AUC: area under the curve; CI: confidence intervals

Similarly, for AUC (0-24) hours, our analysis of the pooled data showed that a significantly higher AUC was achieved with high-dose rifampin compared to standard-dose rifampin (MD 148.66; 95% CI (19.38, 277.94), P=0.02), but a very high level of statistical heterogeneity was observed (I²=95%). A forest plot of these results is shown in Figure 4.

A leave-one-out sensitivity analysis was carried out for both outcomes. For C-max, the results did not change, and heterogeneity remained high. However, upon removing Ruslami et al. (2013) and Yunivita V et al. (2016), the heterogeneity decreased from 95% to 53% for AUC (0-24), and the significance also increased (MD 234.43; 95% CI (149.62, 319.23), P<0.00001).

Six-month mortality

**Figure 5:**
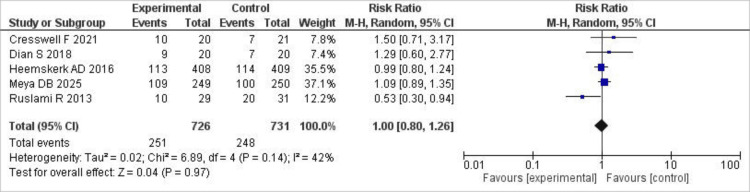
Forest plot of six-month mortality This forest plot illustrates the comparison of six-month mortality between high-dose and standard-dose rifampin groups using RR with 95% CI [7,9,15,17,18]. RR: risk ratios; CI: confidence intervals

Only five studies reported on six-month mortality; however, we observed no significant difference in mortality rates between the groups and only mild statistical heterogeneity (I²=42%; RR 1.00; 95% CI (0.80, 1.26), P=0.97). The results of this outcome are shown in Figure 5.

The sensitivity analysis showed that, after removing Ruslami R 2013 et al., heterogeneity dropped to 0; however, the results remained insignificant (RR 1.07; 95% CI (0.92, 1.24), P=0.37).

Adverse events

**Figure 6:**
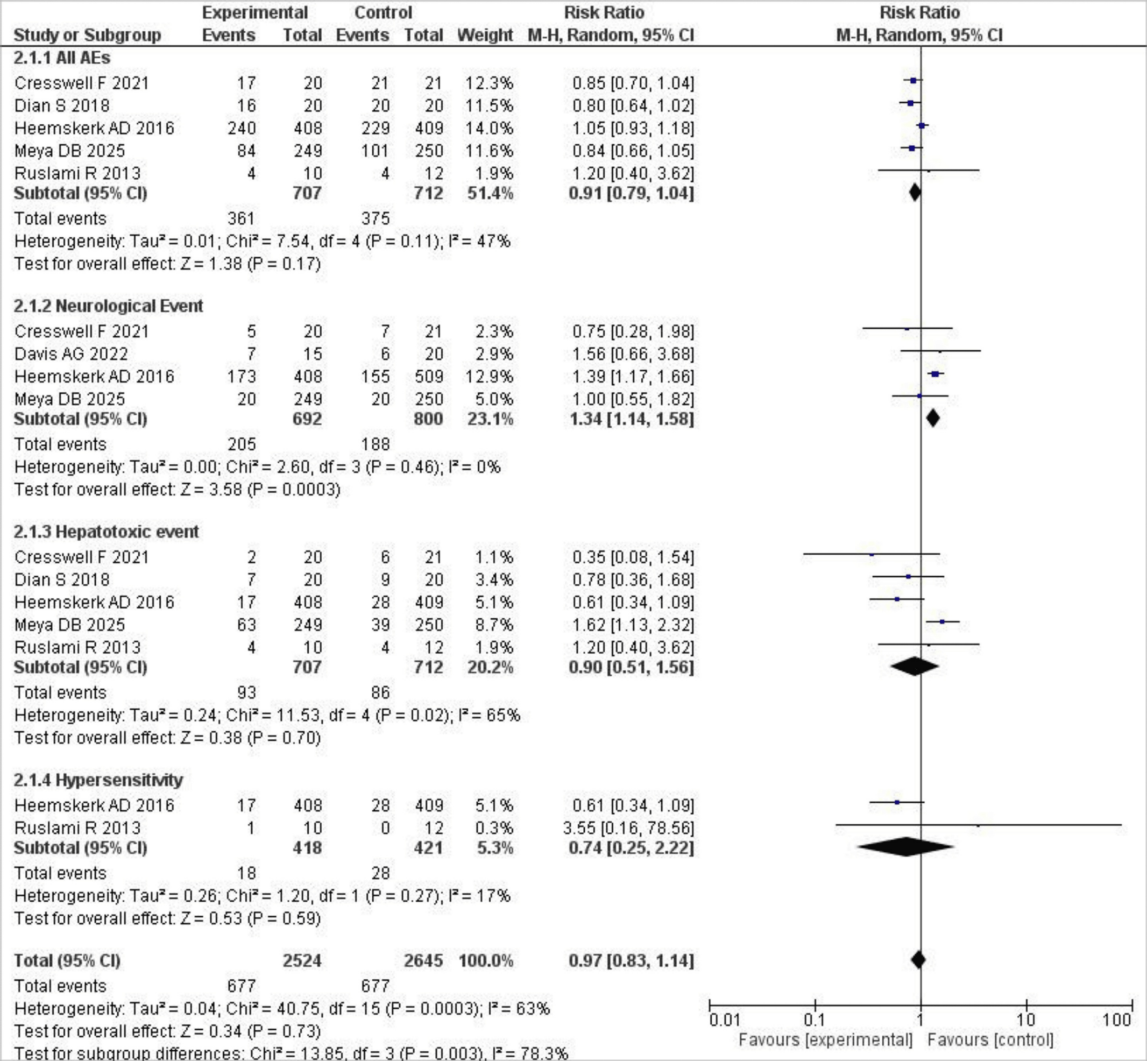
Forest plot of adverse events This figure presents pooled RR for adverse outcomes, including neurological events, hepatotoxicity, hypersensitivity, and overall adverse events between treatment groups [7,9,15,17,18]. RR: risk ratios; CI: confidence intervals

We pooled data on any adverse events, neurological events, hepatotoxic events, and hypersensitivity events. The only significant finding was an increased risk of neurological events with high-dose rifampin compared to standard-dose rifampin (RR 1.34; 95% CI (1.14, 1.58); P=0.003), with no statistical heterogeneity observed. For any adverse events (RR 0.91; 95% CI (0.79, 1.04); P=0.17), hepatotoxic events (RR 0.90; 95% CI (0.51, 1.56); P=0.70), and hypersensitivity events (RR 0.74; 95% CI (0.25, 2.22); P=0.59), the results were not statistically significant. Mild heterogeneity was observed for any adverse events (I²=47%) and moderate heterogeneity for hepatotoxic events (I²=65%). A forest plot illustrating these results is presented in Figure 6.

After excluding Heemskerk et al. from the analysis, the results became significant, and heterogeneity dropped to zero (RR 0.84; 95% CI (0.74, 0.95); P=0.006).

Discussion

This meta-analysis and systematic review assessed the effectiveness and safety of high-dose rifampicin vs. standard-dose rifampicin in the treatment of TBM. Eight RCTs were included, in which baseline characteristics were relatively similar between the intervention and control groups, allowing reliable comparison of treatment outcomes.

In our meta-analysis, high-dose rifampicin was found to significantly increase C-max compared to the standard dose (MD 23.89 µg/mL; 95% CI (8.56, 39.21); P=0.002). This pharmacokinetic benefit is reinforced by a Phase II clinical trial in adults with TBM, in which high-dose oral (35 mg/kg) and intravenous (20 mg/kg) rifampicin demonstrated significantly higher plasma and CSF concentrations with no overt toxicity compared to the conventional 10 mg/kg induction dose. Population pharmacokinetic modelling continues to show that standard doses are often insufficient to achieve therapeutic CSF concentrations, whereas higher doses significantly increase systemic and CNS exposure [6,7].

High-dose rifampicin in our analysis produced a significant difference in total drug exposure (AUC (0-24)) relative to the standard dose (MD 148.66 µg·h/mL; P=0.02), although heterogeneity was high. Similarly, prior pharmacokinetic data have demonstrated that oral doses of 35 mg/kg result in significantly higher AUC than standard dosing and equal or greater exposure than intravenous regimens, thereby demonstrating the pharmacokinetic benefit of high-dose rifampicin in TBM [14,19].

Our leave-one-out sensitivity analysis revealed that high-dose rifampicin significantly increased AUC (0-24), and heterogeneity decreased with the removal of some studies. Pharmacokinetic research in TBM patients has confirmed this observation, showing that higher oral rifampicin doses significantly increase total drug exposure compared with standard dosing and that variability in Cmax between studies reflects differences in administration route and patient factors [14].

High-dose rifampicin was not associated with a significant effect on six-month mortality relative to the standard dose (RR 1.00; 95% CI (0.80, 1.26); P=0.97), with mild heterogeneity. This is in line with other meta-analyses that have found no survival advantage of high-dose rifampicin in patients with TBM, even with higher drug exposures, and no significant change in serious adverse events [20,21].

High-dose rifampicin was associated with a higher risk of neurological adverse events (RR 1.34), but no significant difference was observed in overall adverse events, hepatotoxicity, or hypersensitivity reactions. The results of this study indicate that the two dosing strategies have a similar safety profile, but higher rifampicin doses are associated with an increased risk of neurological complications. These results are largely consistent with earlier meta-analyses, which also found no significant increase in the number of adverse events with increasing rifampicin dose. Nonetheless, our findings indicate an increase in neurological events, suggesting that high-dose rifampicin is not particularly unsafe, although close observation of neurological complications may be justified [8,20]. High-dose rifampicin did not significantly affect the risk of hepatotoxicity (RR 0.90; 95% CI (0.51, 1.56)) or hypersensitivity (RR 0.74; 95% CI (0.25, 2.22)). These results are consistent with prior research, such as a dose-escalation trial in TBM patients, which found no evidence of increased grade 3-4 adverse events with high-dose rifampicin up to 30 mg/kg, and a meta-analysis of 12 RCTs showing similar safety profiles for high- and standard-dose rifampicin [15,22].

In general, the results of this meta-analysis indicate that the relationship between pharmacokinetic optimization and clinical outcomes in TBM is complex. A potential explanation for the lack of observed mortality benefit despite improved pharmacokinetic exposure relates to the pathophysiology of TBM. Most antimicrobial agents, such as rifampicin, have limited penetration across the blood-brain barrier, especially in later stages of treatment when inflammation is reduced. Higher doses of rifampicin can raise plasma and CSF levels, but these changes may not be sufficient to produce optimal bactericidal effects in the central nervous system. Moreover, host inflammatory responses, cerebral oedema, and vascular complications such as infarction also contribute to mortality in TBM, in addition to bacterial burden. Hence, maximum antimicrobial exposure might not significantly impact clinical outcomes without considering the inflammatory aspect of the disease.

Limitations and future directions

There are a few limitations to this study that should be considered when interpreting the findings. To begin with, the RCTs included in the analysis were rather small, which limited the statistical power of some results and precluded the evaluation of publication bias. Second, there was high heterogeneity in pharmacokinetic results, likely due to variations in dosing schedules, routes of administration, study populations, and baseline disease severity across trials. Third, subgroup analyses according to HIV status, disease severity, or route of drug administration were not possible because of the lack of data availability.

Another limitation is that some of the included studies had issues with blinding and randomization, which could introduce bias. Additionally, differences in follow-up and outcome reporting across studies could have affected the pooled estimates. Furthermore, a comprehensive assessment of treatment efficacy is not achievable because long-term neurological outcomes and functional recovery cannot be measured.

Future studies need to be large, well-designed randomized trials of optimized rifampicin dosing regimens with standardized outcomes. Research should also explore personalized dosing strategies based on pharmacokinetic monitoring. In addition, studies on the use of combination therapeutic approaches, such as adjunctive anti-inflammatory or neuroprotective interventions, could enhance patient outcomes in TBM. Exploring patient subgroups, including those with HIV co-infection or severe disease, may further help identify groups that would benefit most from intensified treatment regimens.

## Conclusions

In conclusion, high-dose rifampicin markedly enhances pharmacokinetic exposure in patients with TBM but does not appear to affect mortality. Although the overall safety profile is similar to that of standard dosing, a higher rate of neurological adverse events should be monitored. More extensive RCTs are needed to determine whether optimal rifampicin dosing strategies can improve clinical outcomes in patients with TBM.
